# Pressurized IntraThoracic Aerosol Chemotherapy (PITAC) directed therapy of patients with malignant pleural effusion and pleural metastasis

**DOI:** 10.1515/pp-2024-0008

**Published:** 2024-11-18

**Authors:** Pernille Schjødt Hansen, Martin Graversen, Sönke Detlefsen, Alan Patrick Ainsworth, Michael Bau Mortensen

**Affiliations:** Odense PIPAC Center (OPC) and Odense Pancreas Center (OPAC), Odense University Hospital, Odense, Denmark; Department of Surgery, HPB and Upper GI Section, Odense University Hospital, Odense, Denmark; Department of Pathology, Odense University Hospital, Odense, Denmark; Department of Clinical Research, Faculty of Health Sciences, University of Southern Denmark, Odense, Denmark

**Keywords:** malignant pleural effusion, pleural metastasis, palliative treatment, thoracic regression grading score (TRGS), pressurized intrathoracic aerosol chemotherapy (PITAC)

## Abstract

**Objectives:**

Pressurized IntraThoracic Aerosol Chemotherapy (PITAC) has been suggested as a new therapy for patients with malignant pleural effusion (MPE) and/or pleural metastasis (PLM). The patients have a poor prognosis with a median survival of 3 to 12 months. We present feasibility, patient safety, and cytological/histological response assessment in PITAC-treated patients with MPE and/or PLM.

**Methods:**

Patients eligible for PITAC and treated at Odense PIPAC Center were included. PITAC was performed in lateral decubitus or prone position under double-lumen endotracheal tube ventilation to allow exclusion of the lung if necessary. After positioning of the ultrasound-guided trocar, the second trocar is inserted by video-assisted thoracoscopy. MPE was evacuated and measured. Pleural lavage was performed if no or small amounts of MPE were present. MPE or pleural lavage fluid was analyzed cytologically. Visible PLM was biopsied and sent for histology assessment using a four-tiered Thoracic Regression Grading Score (TRGS). After a walkthrough of the safety checklist, the chemotherapy was nebulized followed by 30 min of passive diffusion. The chemotherapy and chemotherapy-saturated air was evacuated through a closed bag and ventilation system.

**Results:**

We report data on 11 intended PITACs in five patients. Nine PITACs were completed and two PITACs were discontinued due to intraoperative complications or technical reasons. Response evaluation was available in three patients: one showed complete response (TRGS 1) and another stable disease (TRGS 2). Cytology was available from two patients: one showed conversion from malignant to benign. The 30-day mortality was zero.

**Conclusions:**

PITAC appears to be safe and feasible.

## Introduction

Malignant pleural effusion (MPE) is caused by malignant cells altering the equilibrium in parietal and visceral pleura resulting in fluid build-up [1]. Depending on the primary malignancy, median survival ranges from 3 to 12 months indicating advanced disease [2, 3]. Treatment options including talcum pleurodesis, indwelling pleural catheters, and serial ultrasound-guided pleurocentesis are all palliative and focus on relief of symptoms [4, 5].

Fifteen percent of Danish cancer patients suffer from MPE, and MPE accounts for 20 % of the total number of pleural effusions registered in Denmark 5], [6], [7. Pleural metastasis (PLM) develops due to the spread of malignant cells through the bloodstream, lymphatic vessels, shedding, or by direct invasion [8]. PLM is often associated with MPE and is the most frequently recurring metastatic malignancy involving the pleural membranes [9, 10]. Furthermore, PLM is also the second most frequent cause of pleural effusion in adults [8].

Pressurized IntraThoracic Aerosol Chemotherapy (PITAC) was first introduced in 2012 in Herne, Germany. During standard thoracoscopy, antineoplastic agents are nebulized under pressure into the pleural cavity through a CE-certified nebulizer [11, 12]. The PITAC procedure was conceptualized in parallel with Pressurized IntraPeritoneal Aerosol Chemotherapy (PIPAC), which is now used globally in patients with peritoneal metastasis (PM). Preclinical PIPAC studies have shown an increased local drug bioavailability compared to lavage-based treatments [13].

In a recent review, five retrospective case series on PITAC-directed therapy in patients with MPE and/or PLM were identified [14]. It concluded that study populations were heterogeneous with missing data on indication, procedure, patient and personnel health safety, postoperative complications, response assessment, and follow-up.

This study investigates feasibility, safety, and cytological–histological response in patients with MPE and/or PLM treated with PITAC.

## Methods

This is a retrospective analysis of consecutive patients with PLM and/or MPE treated with PITAC at Odense PIPAC Center (OPC) from February 2018 to September 2021. Patients with an Eastern Cooperative Oncology Group (ECOG) performance status ≤2 were included by case-by-case assessment and discussed at the multidisciplinary PIPAC conference. They were informed regarding the experimental nature of PITAC including its potential effects, complications, and adverse events. Additionally, patients were informed about the off-label use of standard chemotherapeutic agents used for PIPAC at OPC since 2015.

### Procedure

**Figure 1:**
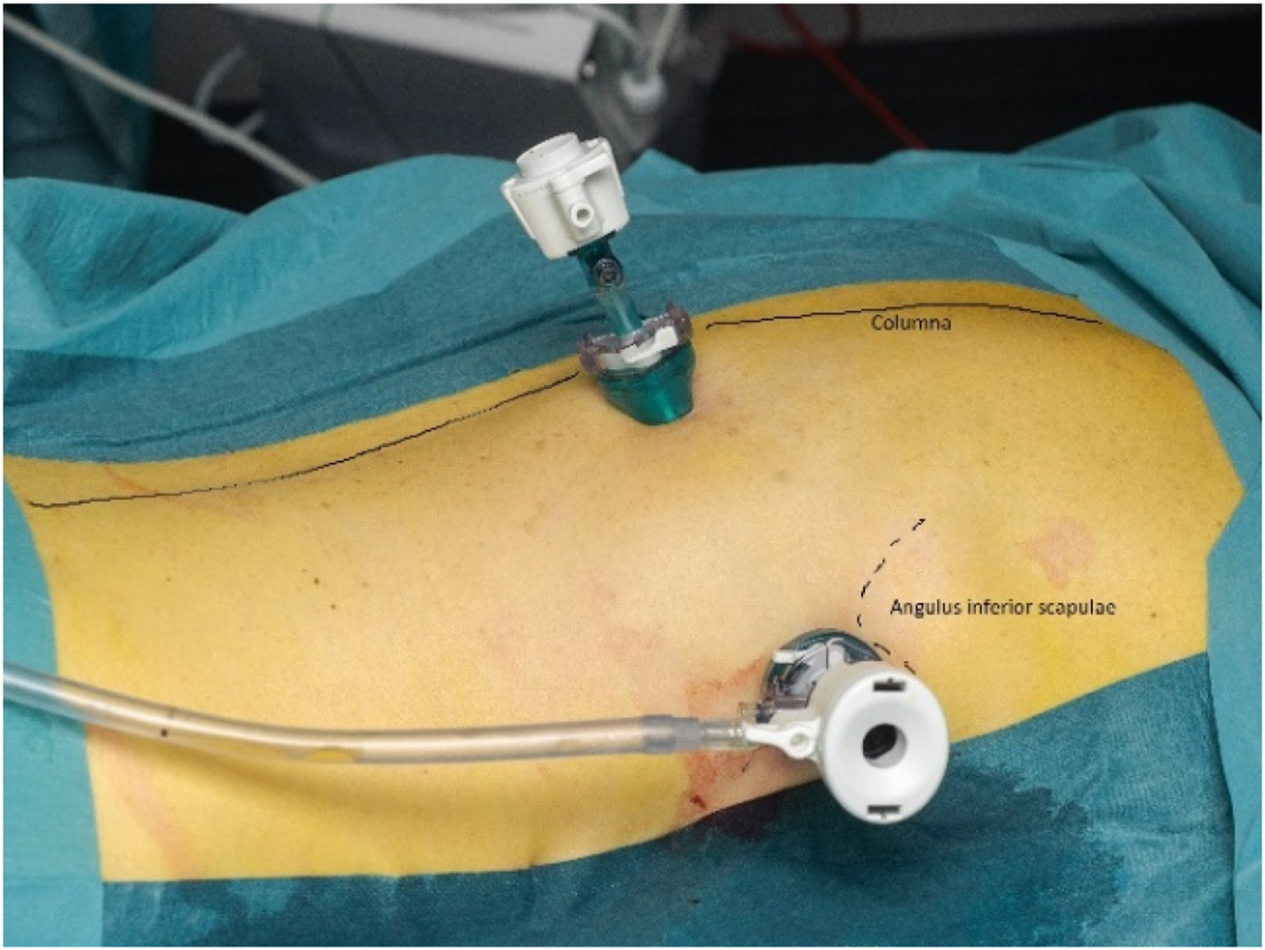
Pressurized IntraThoracic Aerosol Chemotherapy in prone position.

With the patient in either the lateral decubitus or prone position, the procedure was performed in general anesthesia using a double lumen endotracheal tube allowing exclusion of the ipsilateral lung if necessary (Figure 1).

A 12 mm balloon trocar placed in the posterior axillary line just inferior to the inferior angle of scapula, and a 5 mm balloon trocar placed near the spine.

Pleural access was obtained ultrasound-guided along the anterior axillary line (AAL), posterior axillary line (PAL), or midaxillary line (MAL). Intrathoracic pressure was maintained throughout the procedure with normothermic CO_2_ at 12 mmHg.

**Figure 2:**
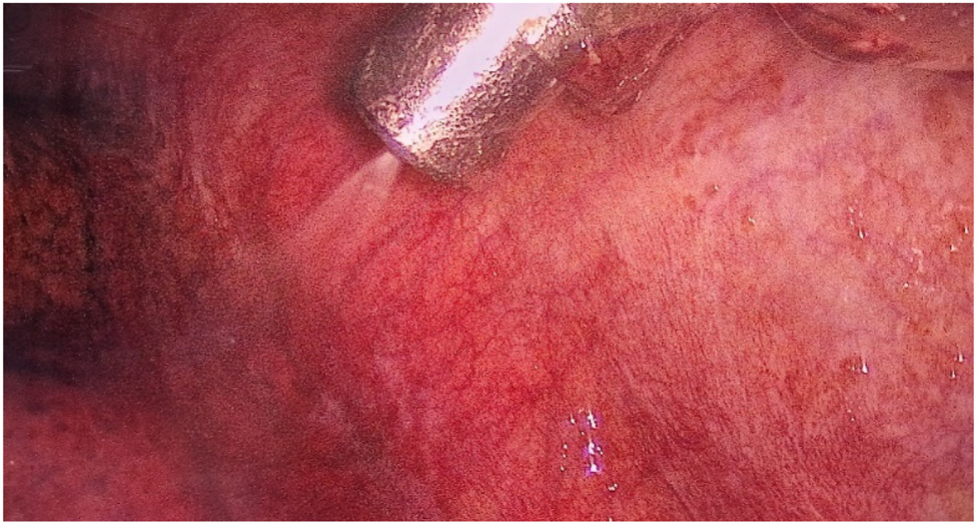
View of the left pleural cavity during nebulization of chemotherapy.

**Figure 3:**
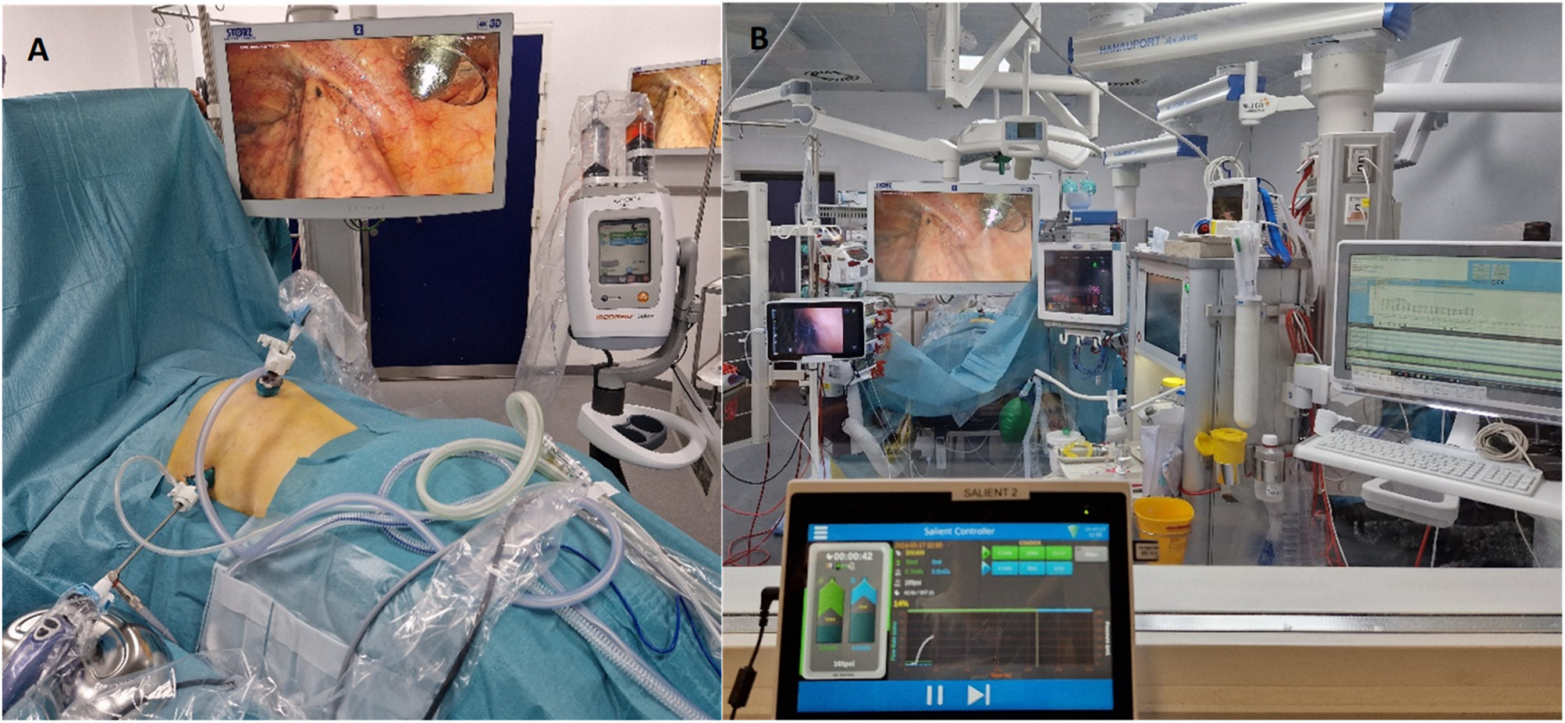
The arrangement during Pressurized IntraThoracic Aerosol Chemotherapy (PITAC) from inside and outside the operating room. (A) The arrangement from inside the operating room with the patient in prone position. Two trocars: 12 mm balloon trocar just next to the spine and a 5 mm trocar below the left scapulae. The left pleural cavity visible with the CE-certified nebulizer in the 12 mm balloon trocar. Microinjector loaded with cisplatin and doxorubicin connected to the CE-certified nebulizer covered by plastic bag. (B) The arrangement from outside the operating room. The following monitors are visual from the left: video-assisted double-lumen endotracheal tube with a view of the tracheal bifurcation, left pleural cavity with the active CE-certified nebulizer, and three monitors related to the anesthesiology. In the front, the remote-control tablet for the microinjector with increasing pressure during nebulization.

After access to the pleural cavity, MPE was evacuated. The pleural cavity was flushed with saline (pleural lavage fluid, PLF) if less than 150 mL of MPE was present. MPE or PLF was analyzed cytologically. If present, parietal PLMs were biopsied and marked by clips, and a CE-certified nebulizer was inserted through the 12 mm trocar (Figures 2 and 3).

A safety checklist, adapted from the OPC standard PIPAC checklist (see Supplemental Material, Appendix 1), was completed before operating room (OR) staff left the OR [15]. The nebulization process was controlled from outside the room with a view of the patient and observation monitors (Figure 3). PITAC was performed using a standard pressure of maximum 300 Psi and a flow rate of 0.5–0.7 mL/s. After 30 min of passive diffusion, the chemotherapy and chemotherapy-contaminated carbon dioxide was evacuated through a closed bag and ventilation system. No chest tubes were inserted. The lung was reventilated under video-guidance when relevant, and the trocar sites closed according to standard thoracoscopy guidelines. The patients received a single prophylactic dose of cefuroxime (3 g) and metronidazole (1.5 g).

Based on chemotherapy applied during PIPAC, patients with colorectal (incl. appendix) cancer received oxaliplatin 92 mg/m^2^. Remaining patients received a combination of cisplatin 7.5 mg/m^2^ and doxorubicin 1.5 mg/m^2^ [16].

### Histology and cytology

MPE cytology, PLF cytology, and pleural biopsies were assessed by the same pathologist (SD) who had experience with evaluation of the Peritoneal Regression Grading Score (PRGS) used in PIPAC-directed therapy [17, 18].

Three step sections were cut from the paraffin embedded tissue blocks and stained with hematoxylin–eosin (H&E), followed by a section immunostained for epithelial cell adhesion molecule (Ep-CAM) or, in case of malignant mesothelioma, Hector Battifora and MEsothelioma 1 (HBME1) [19]. A final series of three step sections were stained with H&E. In analogy with the PRGS, a four-tiered Thoracic Regression Grading Score (TRGS) was assessed: TRGS one equaled complete response, TRGS two equaled major response, TRGS three equaled minor response, and TRGS four equaled no response. If more than one pleural biopsy was obtained, the mean TRGS was also reported.

MPE and/or PLF were analyzed as described previously [20]. A minimum of 150 mL of MPE and/or PLF was centrifuged, and smears of the sediment were analyzed by conventional cytology (Papanicolaou and May-Giemsa Grünwald staining). Leftovers of the sediment were embedded in paraffin wax of which one section was stained with H&E. If necessary, further sections were analyzed with immunocytochemical markers such as calretinin, cluster of differentiation (CD) 56, carcinoembryonic antigen (CEA), cytokeratin (CK) 7, CK20, Ep-CAM, HBME-1, protein ΔNp63 (P40), synaptophysin, or thyroid transcription factor 1 (TTF1), as part of the diagnostic routine. The cytological specimens were diagnosed using a five-tiered system: malignant cells, cells suspicious of malignancy, atypical cells, no malignant cells, or other (including specimens unsuitable for diagnosis for technical reasons).

### Assessment of complications

Patients were evaluated by PITAC surgeons before discharge. A hotline phone number was provided at discharge, and the patients were contacted after 2 weeks.

Surgery-related complications were graded according to the Clavien–Dindo Classification (grade I–V) [21], and PITAC-related adverse events according to the Common Terminology Criteria for Adverse Events (CTCAE) version 4.0 [22].

## Results

### Procedures

**Figure 4:**
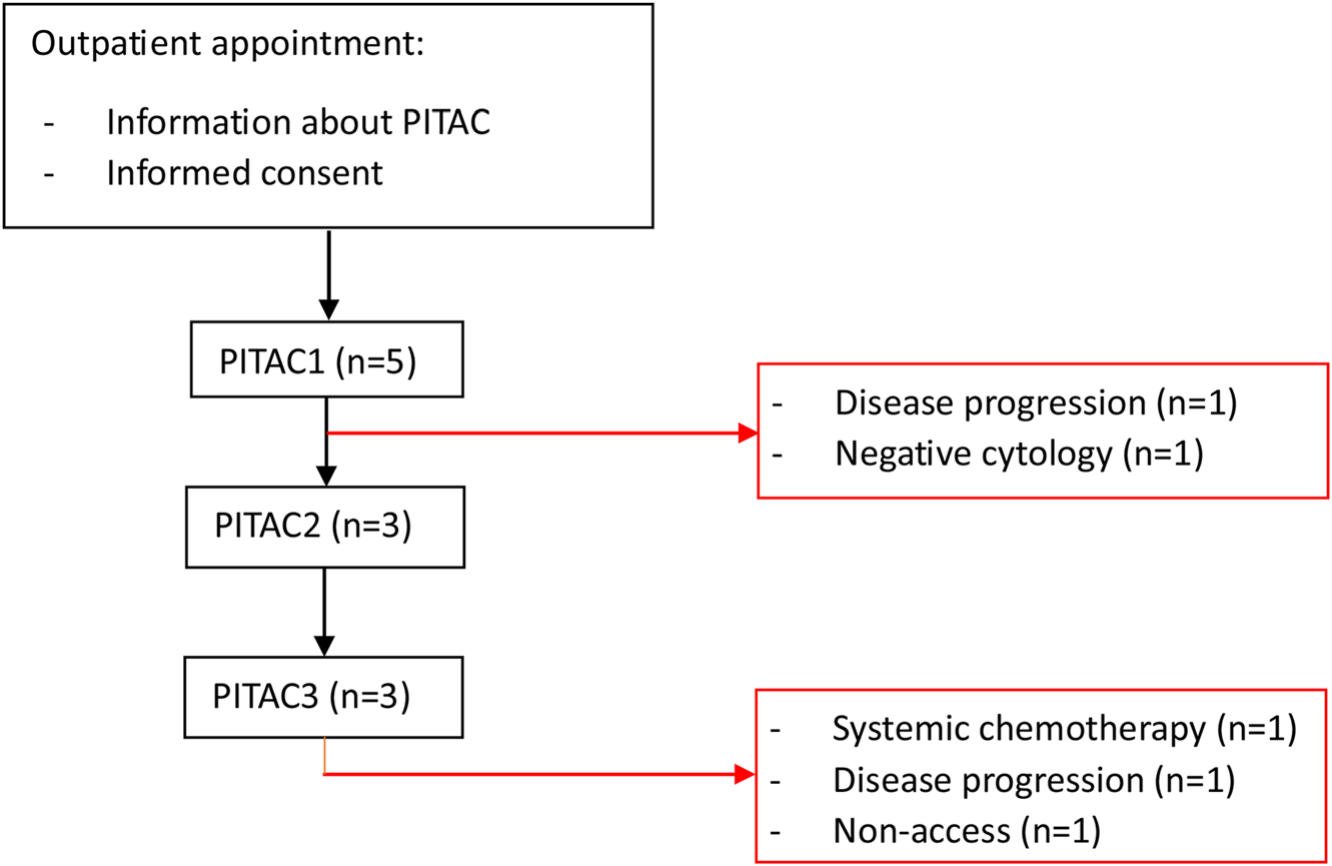
Patient flowchart.

Five patients were found eligible for PITAC-directed therapy (Figure 4). Indications for PITAC were MPE (n=2), PLM (n=1), or synchronous MPE and PLM (n=2) from malignant mesothelioma or metastasis from carcinoma of different origin (rectum, breast, ovary, or stomach) (Table 1).

**Table 1:** Patient characteristics.

Patient (primary)	1 (Rectum)	2 (Breast)	3 (Ovary)	4 (Peritoneum)	5 (Stomach)
Gender, F/M	M	F	F	M	M
Age, years	53	56	67	61	64
ECOG performance status	0	1	0	0	2
Histology	Adenocarcinoma	HER2+ ductal carcinoma	High-grade serous adenocarcinoma	Malignant epithelioid mesothelioma	Poorly cohesive (signet ring cell) carcinoma
Other metastatic sites	No	No	No	No	No
Prior treatment	Systemic chemotherapy Lung resection SBRT	Systemic chemotherapy Left-sided mastectomy Immunotherapy Herceptin treatment	Systemic chemotherapy HIPEC Immunotherapy	Systemic chemotherapy	Gastrectomy Systemic chemotherapy
Bidirectional treatment	Systemic chemotherapy	Immunotherapy	None	None	None
Indication for PITAC	Left-sided PLM	Bilateral MPE and PLM	Left-sided MPE^a^	Right-sided MPE and PLM	Left-sided MPE
Days between MPE/PLM diagnosis and PITAC1	843	108	873	N/A	40

^a^Indication was malignant pleural effusion (MPE), but both MPE and pleural metastasis (PLM) were found at PITAC1. ECOG, Eastern Cooperative Oncology Group; HER2+, human epidermal growth factor receptor two positive; SBRT, stereotactic body radiotherapy; HIPEC, heated intraperitoneal chemotherapy; N/A, not available.

Patients 1–3 received two or more PITACs and patient 4 received one PITAC (Table 2). For patient 5, two PITACs were planned but terminated due to an intraoperative complication during PITAC1 and later disease progression.

**Table 2:** Procedures and outcome.

Patient (primary)	1 (Rectum)	2 (Breast)	3 (Ovary)	4 (Peritoneum)	5 (Stomach)
Positioning	Prone	PITAC1+2: Lateral PITAC3: Prone	Lateral	Prone	Prone
Balloon trocar positioning	N/A	PAL IC7 and caudal AAL	MAL IC5-6 and caudal PAL/MAL IC-7-8	PAL below the scapula and just right to the spine	PAL below the scapula and just right to the spine
No. of PITACs	3	3	3	1	1
MPE volume, mL	PITAC1: 50 PITAC2: 50 PITAC3: Non-access	PITAC1: 1,250 PITAC2: 1,250 PITAC3: 1,100	PITAC1 (lhs): 30 PITAC2 (lhs): 0 PITAC3 (rhs): 700	PITAC1: 400	PITAC1: N/A
Pleural lavage	PITAC1: Yes PITAC2: Yes PITAC3: Non-access	PITAC1: No PITAC2: No PITAC3: No	PITAC1 (lhs): Yes PITAC2 (lhs): Yes PITAC3 (rhs): No	PITAC1: No	PITAC1: No
Cytology	PITAC1: Malignant cells PITAC2: Not suited for diagnosis PITAC3: NA	PITAC1: Malignant cells PITAC2: No malignant cells PITAC3: No malignant cells	PITAC1 (lhs): Malignant cells PITAC2 (lhs): Malignant cells PITAC3 (rhs): Malignant cells	PITAC1: Atypical cells	PITAC1: NA
Histology, TRGS^a^	PITAC1: 3.0 (2 biopsies) PITAC2: 1 (1 biopsy) PITAC3: NA	PITAC 1: NA PITAC 2: NA PITAC 3: NA	PITAC1 (lhs): NA PITAC2 (lhs): 2 (1 biopsy) PITAC3 (rhs): 2 (1 biopsy)	PITAC1: 1 (1 biopsy)	PITAC1: NA
Concurrent surgeries	No	PIPAC with all PITACs	One PIPAC at PITAC3	One PIPAC at PITAC1	No
Intraoperative complications (Clavien-Dindo)	Intercostal arterial bleed during biopsy (IIIb)	None	None	None	Lung lesion during access
Postoperative complications (CTCAE grade)	PITAC1: Chest pain (2) PITAC2: Vomiting (2), chest pain (2) PITAC3: None	PITAC1: Chest pain (2) and urinary retention (1) PITAC2: Chest pain (2), subcutaneous emphysema (1), and urinary retention (1) PITAC3: Chest pain (2)	None	PITAC1: Chest pain (2) and urinary retention (2)	PITAC1: Subcutaneous emphysema (1)
Length of stay, days	PITAC1: N/A PITAC2: 1 PITAC3: 0	PITAC1: 1 PITAC2: 1 PITAC3: 1	PITAC1 (lhs): 1 PITAC2 (lhs): 0 PITAC3 (rhs): 0	PITAC1: 1	PITAC1: 1

^a^In case of several biopsies taken prior to the same PITAC, mean Thoracic Regression Grading Score (TRGS) is given. ^b^Control X-ray due to insufficient reventilation of the right lung due to PLM., AAL, anterior axillary line; CTCAE, Common Terminology Criteria for Adverse Events; lhs, left-hand side; MAL, midaxillary line; NA, not available; PAL, posterior axillary line; rhs, right-hand side.

### Response assessment

#### Cytology

Pleural cytology was available in patients 1–4 (Table 2). For patient 1, malignant cells were found in the specimen taken prior to PITAC1, while the specimen from PITAC2 was inconclusive for technical reasons. Patient 2 underwent three PITACs, and malignant cells were detected in the specimen obtained prior to PITAC1 but no longer detectable prior to PITAC2 and -3. For patient 3, malignant cells were found in three consecutive specimens taken prior to PITAC1, -2, and -3. No comparison was possible for patient 4 since only one conclusive cytology specimen was available, showing atypical cells, why no additional PITACs were performed.

#### Histology

**Figure 5:**
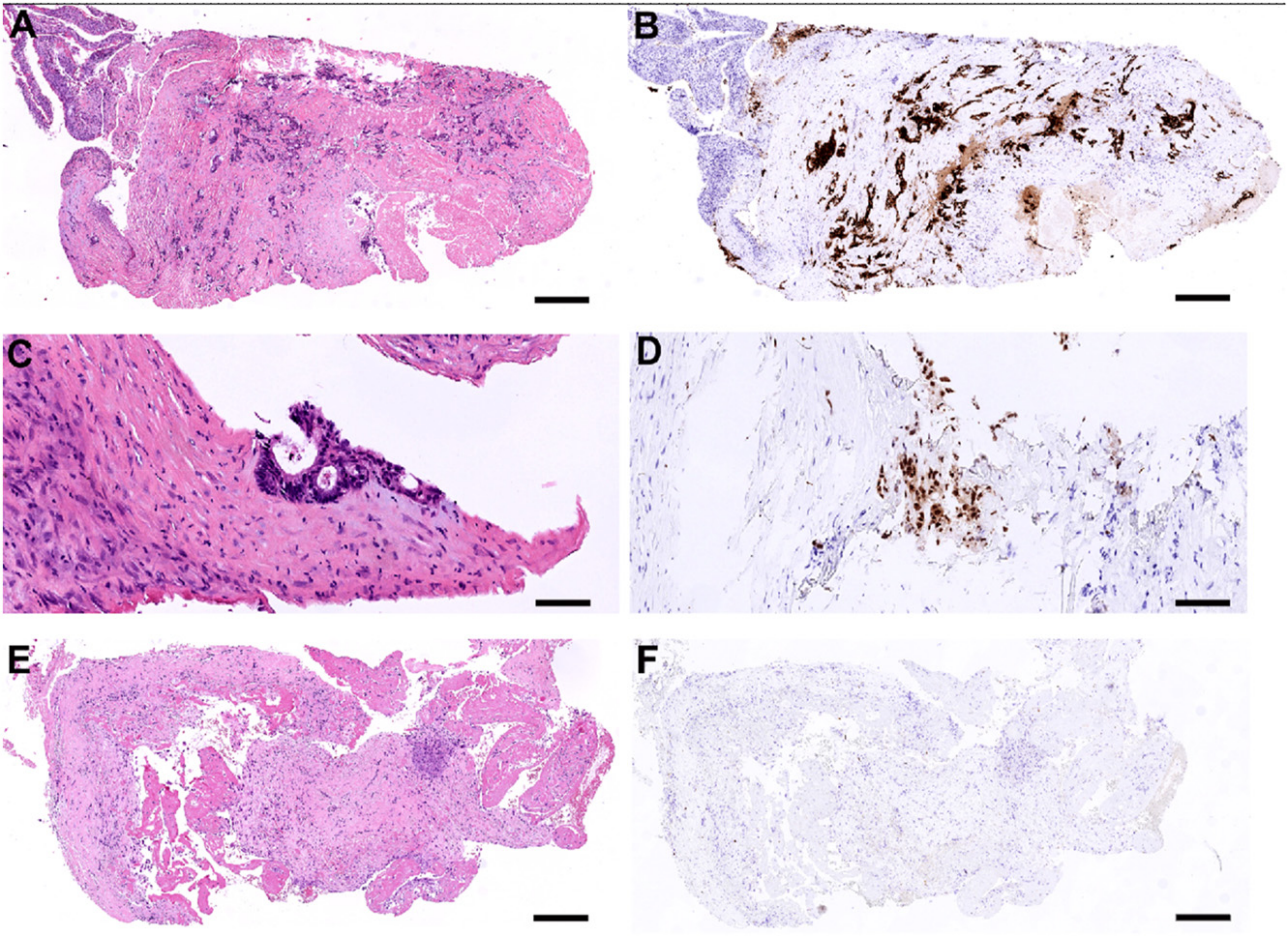
Histological features of pleural metastasis from rectal cancer before and after PITAC (patient 1). (A and B) Biopsy from the upper left-sided pleura at baseline, showing metastatic rectal adenocarcinoma, TRGS 4 (a, H&E. B, CDX2-immunostaining). Scale bar: 500 μm. (C and D) Higher magnification of biopsy from the lower left-sided pleura at baseline, showing a small focus of metastatic rectal adenocarcinoma, TRGS 2 (C, H&E. D, CDX2-immunostaining). Scale bar: 100 μm. (E and F) Biopsy from the left pleura taken prior to PITAC2, showing complete response (TRGS 1). Regressive fibrosis with mesothelial proliferation and fibrin is shown. No metastatic tumor cells are found at this location (E, H&E. F, CDX2-immunostaining). Scale bar: 350 μm.

Biopsies were available from patient 1, 3, and 4 (Table 2). In patient 1, the mean baseline TRGS, prior to PITAC1, was 3.0 (Figure 5a–d). Prior to PITAC2, the biopsy showed complete regression (TRGS 1) (Figure 5e and f). Patient 3 had no biopsies taken at baseline, while biopsies taken prior to PITAC2 (left pleural cavity) and prior to PITAC3 (right pleural cavity) showed TRGS 2. Patient 4 showed TRGS one at baseline, prior to PITAC1, while no consecutive biopsies were available.

#### Intra- and postoperative complications

Patient 5 scheduled for PITAC did not receive PITAC because of a lung lesion during access (uneventful postoperative recovery). Patient 1 had an intraoperative hemorrhage caused by the pleural biopsy at PITAC2, but this did not influence treatment. No intraoperative events that may have posed a risk to the occupational health safety were registered.

Mild to moderate (CTCAE≤2) medical AEs were reported by three patients (Table 2). Patient 1 had vomiting and chest pain after PITAC1 and -2. Patient 2, who received simultaneous PIPAC-directed therapy, had chest pain and urinary retention after PITAC1 and -2. The same patient had right-sided self-limiting subcutaneous emphysema. Patient 4 had mild chest pain and urinary retention after PITAC1.

No severe or life-threatening (CTCAE>2) postoperative medical AEs were reported. All patients were either discharged the same day or on the first postoperative day.

#### Follow-up

Based on CT scans, patient 1 showed stable disease for 11 months. Patient 2 had CT-verified disease progression within 6 months. Patient 3 showed no recurring MPE on the left-hand side during a lung ultrasound 4 weeks after PITAC2. Six weeks after PITAC2, a CT-scan showed small amounts of MPE bilaterally. Patient 4 had no evidence of malignant disease after PITAC1.

## Discussion

In this retrospective analysis of patients with MPE and/or PLM treated with PITAC from 2018 to 2021, five patients were included on a case-by-case assessment, resulting in 11 intended PITAC treatments of which nine were completed. The included patients were heterogeneous in characteristics, including primary tumors and performance status. The use of PITAC has previously been reported in patients with malignant mesothelioma, ovarian, and gastric cancer [12, 23, 24]. To our knowledge, this is the first report of pleural metastasis from rectal and breast cancer treated with PITAC.

Two intraoperative complications were registered: one lung lesion during port insertion in a patient with left-sided MPE from gastric cancer and one intercostal arterial bleed during a biopsy in a patient with left-sided MPE from rectal cancer. In the first patient, it was assessed that it would not be safe to proceed with PITAC, but in the second case, PITAC was administered after hemostasis. Previous studies on PITAC have not reported intraoperative complications [12, 23], [24], [25], [26.

We identified two cases of CTCAE≤2. One study found no CTCAE>2 related to PITAC; however, CTCAE<2 was published as a mixture of PIPAC and PITAC data [24]. Another study found prolonged air-leakage in a patient with simultaneous lung wedge resections. Other possible complications such as toxic reactions or wound heling problems could not be determined, as data were a mixture of PIPAC and PITAC [25]. Thus, results on intra- and postoperative complications are lacking.

The most useful histological response data available from this study was from patient 1 with PLM from rectal adenocarcinoma. The patient was planned to receive three PITACs – two were completed and the third was with primary non-access. The biopsies showed complete regression from mean TRGS 3.0 prior to PITAC1 to TRGS 1 prior to PITAC2, indicating a local histological response. From patient 3 (PLM from ovarian cancer), pleural biopsies taken prior to PITAC2 and -3 both showed TRGS 2 (major histological response). Pre- and post-treatment biopsies for patients 2, 4, and 5 were unfortunately not available.

The use of the TRGS, in analogy to the PRGS in PM, is supported by recent data showing that the PRGS holds significant prognostic value in patients treated with PIPAC-directed therapy [16]. The present data suggest that PITAC may induce histological regression in PLM based on the TRGS, but at present, no other data are available. Further prospective data are needed to establish the utility of TRGS in the setting of PITAC further, and particularly its potential clinical impact.

Cytology for response evaluation of PITAC was not used previously, according to the published literature [12, 23], [24], [25], [26. This retrospective study found conversion in one patient with right-sided MPE from HER2+ ductal breast cancer. Before PITAC1, malignant cells were detectable in the MPE. At PITAC2 and -3, no malignant cells were detectable indicating that PITAC might have a positive effect on MPE in breast cancer patients. However, as breast cancer has never before been treated with PITAC and only one patient was included in this study, further studies are needed to support these results.

There are no data comparing PITAC to other available treatment options in patients with MPE such as talcum pleurodesis, indwelling pleural catheters, and repeated ultrasound-guided pleurocentesis. PITAC is performed under general anesthesia demanding that patients should be in good performance and this might limit some patients from PITAC compared to the conventional treatment options for MPE. Still, one very important feature with PITAC is the use of chemotherapy. The current treatment options focus on symptomatic relief only by removing the MPE and/or gluing the pleura together with talcum, but by applying chemotherapy to the pleural cavity, local treatment of malignant cells and/or visible PLM is also provided.

Whether PITAC is more or less safe and effective than the current treatment options cannot be determined based on the present study. However, it is interesting to note that PITAC-directed therapy seems technically feasible and may induce local response when evaluated in the same way as patients having PIPAC [12, 23], [24], [25], [26.

The advancement of PITAC since 2012 has been slow-paced, as merely 21 published patients have received a total of 38 PITACs [12, 23], [24], [25], [26. Five retrospective case series (six including the present) classify PITAC to phase-I of the IDEAL framework [27]. In May 2023, the first prospective phase-I study was approved at Odense PIPAC Center, Denmark, and patients are currently being recruited. The primary goal is to evaluate feasibility and safety of PITAC-directed therapy in patients with MPE, while secondary outcomes include MPE volume reduction and histological/cytological response [28].

## Conclusions

PITAC seems technically feasible, safe, and able to induce histological and cytological response in some patients with MPE and/or PLM. However, prospective phase-I data are needed to investigate standard operating procedures, indication, patients and occupational health safety, optimal response assessment, and subsequently relevant short- and long-term outcomes.

## Supplementary Material

Supplementary Material
