# Supplementary material for: Pressurized IntraThoracic Aerosol Chemotherapy (PITAC) directed therapy of patients with malignant pleural effusion and pleural metastasis
Source: Pleura Peritoneum. 2024 Nov 18;9(4):131–9. doi: 10.1515/pp-2024-0008 (PMC11661464; doi:10.1515/pp-2024-0008)
Supplement: Supplementary file 1 — Supplementary Material [file j_pp-2024-0008_suppl_001.docx]

# PITAC checklist

Patient label:

PITAC procedure no.:

Date:

| Procedure | Yes | No | Comments |
| --- | --- | --- | --- |
| 1. WHO Safe surgery checklist documented? |  |  |  |
| 1. Correct chemotherapy type, dose and patient ID? |  |  |  |
| 1. Spill Kit available? |  |  |  |
| 1. All medical staff wear glasses or other kind of eye protection? |  |  |  |
| 1. Surgeons and scrub nurses wear double layered gloves and protective barrier garments? |  |  |  |
| 1. Floor beneath injector covered by non-permeable blanket? |  |  |  |
| 1. Container for hazardous waste in place? |  |  |  |
| 1. Exsufflation kit with two micro particle filters inserted between the 5 mm trocar and the closed main ventilation system? |  |  |  |
| 1. 5 mm trocar closed? |  |  |  |
| 1. Thermoflator activated with heated CO_2_? |  |  |  |
| 1. Intrathoracic pressure of 12 mmHg? |  |  |  |
| 1. CO_2_ flow rate < 0,2 liters/minute? |  |  |  |
| 1. Subcutaneous emphysema present? |  |  |  |
| 1. PLM score and malignant pleural effusion volume documented in REDCap? |  |  |  |
| 1. Pleural lavage performed? |  |  |  |
| 1. Pleural biopsies taken? |  |  |  |
| 1. CE-certified nebulizer flushed with saline? |  |  |  |
| 1. CE-certified nebulizer inserted and fixated through the 12 mm trocar pointing away from lung tissue? |  |  |  |
| 1. High pressure line tightly connected to the syringe and CE-certified nebulizer? |  |  |  |
| 1. High pressure line covered by plastic bag? |  |  |  |
| 1. Injector settings with a maximum of 300 PSI and flow-rate of 0.5-1.8 ml/sec? |  |  |  |
| 1. Remote control to the injector present? |  |  |  |
| 1. Monitors visual through the window? |  |  |  |
| 1. Anesthesiology - ready for treatment? |  |  |  |
